# Patient perceptions of co-morbidities in inflammatory arthritis

**DOI:** 10.1093/rap/rkaa076

**Published:** 2021-01-11

**Authors:** Gouri M Koduri, Nicola J Gullick, Fiona Hayes, Shirish Dubey, Chetan Mukhtyar

**Affiliations:** 1 Rheumatology Department, Southend University Hospital, Westcliff-on-Sea; 2 Rheumatology Department, University Hospitals Coventry & Warwickshire NHS Trust, Coventry; 3 Rheumatology Department, Norfolk and Norwich University Hospital, Norwich, UK

**Keywords:** co-morbidities, Charlson co-morbidity, inflammatory arthritis, polypharmacy, patient perception, education and outcomes

## Abstract

**Objective:**

Longer life expectancy has resulted in people living with an increasing number of co-morbidities. The average individual with inflammatory arthritis has two co-morbidities, which contribute to higher mortality, poorer functional outcomes and increased health-care utilization and cost. A number of studies have investigated the prevalence of co-morbidities, whereas this study was designed to look at patient perspectives.

**Methods:**

The study comprised two parts: a patient questionnaire and an interview. Individuals with physician-verified inflammatory arthritis along with one or more Charlson co-morbidities were invited to participate. In-depth data were obtained by interviews with 12 willing participants.

**Results:**

One hundred and forty-six individuals were recruited; 50 (35%) had one co-morbidity, 69 (48%) had two and 25 (17%) had more than four co-morbidities. Seventy-seven individuals (53%) reported that co-morbidities affected their health as much as their arthritis, and 82 (56%) reported dependence on others for activities of daily living. Lack of education was highlighted by 106 (73%) participants. Qualitative data provided further support for the challenges, with participants highlighting the lack of time to discuss complex or multiple problems, with no-one coordinating their care. This, in turn, led to polypharmacy and insufficient discussion around drug and disease interactions, complications and self-help measures.

**Conclusion:**

This study highlights the challenges for individuals with inflammatory arthritis who suffer with multiple co-morbidities. The challenges result from limited resources or support within the current health-care environments. Individuals highlighted the poor quality of life, which is multifactorial, and the need for better educational strategies and coordination of care to improve outcomes.

Key messages

- Individuals with inflammatory arthritis can have multiple co-existing conditions with different levels of severity, which need to be taken into account.
- Individuals with inflammatory arthritis reported a range of perceptions about co-morbidities and support preferences.
- Recommendations made by the individuals in this study align with EULAR guidelines on education.

## Introduction

With advances in treatments, remission and improved survival are not only an achievable goal, but a quality target. As a result of living longer and suffering with a chronic inflammatory disease, individuals with inflammatory arthritis have a higher risk of co-morbidities, such as cardiovascular diseases, osteoporosis, depression, infections and cancer [1–4]. Currently, the average sufferer of inflammatory arthritis has two or more co-morbid disorders, which contribute to a higher mortality [2, 5–12]. Improvements in pharmacotherapy result in better functional outcome owing to improved disease control, but co-morbidities might reverse this benefit [13]. Higher numbers of co-morbidities result in greater utilization of health-care resources [14]. RA is also known to result in greater societal costs [15, 16], estimated as €780 million per year in England [17].

Treatment of co-morbidities is challenging. Current guidelines for managing rheumatic diseases do not consider health economics, patient perspectives and the medical impact of the inevitable polypharmacy and interacting medical conditions. There are some data on measurement of co-morbidity and impact on outcomes [2, 4, 7, 13, 14]. However, there is little evidence regarding patient perspectives about living with multiple conditions or how their perspectives relate to professional concepts. If a health-care system is to provide holistic care, we need a better understanding of these relationships. The aim of this study was to explore the individual experience and understanding of co-morbidities and to understand the impact of these co-morbidities on quality of life and activities of daily living.

## Methods

### Setting

Prospective recruitment was carried out in three UK Rheumatology departments: Southend University Hospital, Norwich and Norfolk University Hospital NHS Foundation Trust and University Hospital Coventry & Warwickshire Hospitals NHS Trust.

### Participants

Individuals with a physician-verified diagnosis of inflammatory arthritis and with one or more Charlson co-morbidity were identified by clinicians from routine outpatient clinic attendances. Recruitment commenced in June 2018 and was restricted to an estimated 150 participants, because of expected data saturation.

### Questionnaire

After informed written consent, the participants were invited to complete a questionnaire either at home or in the outpatient department. The survey comprised 26 questions divided into four broad themes (Supplementary Data S1, available at *Rheumatology Advances in Practice* online): demographics, disease-related factors and Charlson co-morbidities; awareness and impact of co-morbidities on quality of life; polypharmacy and treatment perception; and lifestyle.

### Interviews

In the survey, the participants were invited to express willingness to participate in a semi-structured interview. Twelve participants were chosen deliberately (to include demographics, conditions and co-morbidities) to collect complementary in-depth information. We stopped the interviews when thematic saturation was achieved. The interview questions were open ended and covered topics including the impact of Charlson co-morbidities on their quality of life, strategies adopted to manage multiple conditions, patient experience of polypharmacy, barriers to exercise and lifestyle changes, and expectation of services (Supplementary Data S2, available at *Rheumatology Advances in Practice* online). The questionnaire items were derived from discussion, literature review and discussion with patient representatives from a national patient charity.

All interviews were digitally audiotaped, with interviews lasting between 45 and 60 min. Interviewees were also given the time to provide additional comments they thought were relevant to the topics discussed. Interviews were transcribed verbatim by an independent transcribing company (Transcription agency LLP, Hythe, Kent, UK). Data were analysed using a combination of inductive and deductive techniques. Initial analytical summaries of the interviews were organized by labelling data relevant to each of the questions in a thematic approach (Supplementary Data S3, available at *Rheumatology Advances in Practice* online). This was undertaken independently by G.M.K., F.H. and N.J.G. Any discrepancies in coding were resolved by discussion. Similar concepts brought up by different participants were studied in greater detail, leading to the identification of key themes. Individual recommendations on service improvement were also elicited.

### Ethics

Ethical approval for this study was granted by the North London Research Ethics Committee (REC reference: 18/LO/0409), and all participants gave written informed consent.

### Statistical analysis

Data were analysed using Excel. Categorical data are summarized as counts and percentages, and age is presented as the mean and median.

## Results

One hundred and forty-six of 194 invited individuals completed the questionnaire (75% response rate). One hundred and four (71%) had RA, 30 (21%) had PsA, and 12 (8%) had axial spondyloarthritis. Demographics and disease characteristics are summarized in Supplementary Tables S1 and S2, available at *Rheumatology Advances in Practice* online. Fifty (35%) had one Charlson co-morbidity, 69 (48%) had two, and 25 (17%) had more than four Charlson co-morbidities. The most common co-morbidities were hypertension [75 (51%)], pulmonary disease [49 (34%)], diabetes mellitus [35 (24%)], ischaemic heart disease [21 (14%)], cancer [20 (14%)] and transient ischaemic attack [11 (8%)]. Eighty-four (58%) of the respondents were >60 years old. Most respondents were retired [85 (58%)], and 31 (21%) had to cut down working hours because of the co-morbidities. The majority of respondents self-reported the severity of rheumatic disease as either moderate [61 (42%)] or severe [35 (24%)].

### Awareness and impact of co-morbidities

Seventy-seven (53%) reported that Charlson co-morbidities affected their health as much as their arthritis. Seventy (48%) said that they received detailed information about arthritis/Charlson co-morbidities (Supplementary Table S3, available at *Rheumatology Advances in Practice* online). Seventy-two (49%) of the cohort were not aware that Charlson co-morbidities can be caused by medications or complications of arthritis. Eighty-two (56%) of the respondents were more or less dependent on their family members or carers for their activities of daily living. Of those, three-quarters needed help at least twice a week.

### Current therapy, efficacy and their involvement in treatment decisions

Overall, 48 (33%) used an NSAID for arthritis, and 73 (50%) used other analgesics, such as paracetamol, opiates or neuromodulators. One hundred and four (71%) used DMARDs, 41 (28%) biologics and 32 (22%) CSs. One hundred and three (71%) were taking medications for other co-morbid conditions (Supplementary Table S4, available at *Rheumatology Advances in Practice* online). Most respondents [100 (68%)] rated treatment efficacy as sufficiently effective (i.e. improvement), and consistent with this, 43 (29%) reported remarkable improvement and 57 (39%) some improvement. Only six (4%) reported worsening of their inflammatory arthritis. In 17 (12%) individuals, arthritis improved but they developed side-effects, and 7 (5%) required medications to counteract those side-effects. The overall involvement of respondents in their treatment decisions was 107 (73%). Thirty-five (24%) felt that the involvement was minimal. Thirty-two (22%) felt that they were insufficiently, but somewhat, involved in decision-making, and 38 (26%) reported no involvement at all. In contrast, 39 (27%) reported feeling ‘very much’ involved in treatment decisions. One hundred and sixteen (80%) of the respondents said the doctors reviewed their medications at regular follow-ups and had discussed benefits and risks of each treatment. The majority were compliant with arthritis and non-arthritis medications.

### Lifestyle advice and benefits of lifestyle changes

Lack of education was reported by 106 (73%) (Supplementary Table S5, available at *Rheumatology Advances in Practice* online). Also, 43 (30%) individuals had not received any advice on BMI and were not aware of associations between obesity and OA or diabetes, hypertension or heart disease. Likewise, 17 (12%) had not received advice on alcohol and its ill effects. Ninety-five (65%) said they were advised about health benefits of exercise, but 31 (21%) did not exercise in any form. The main reasons for not taking exercise were pain, fatigue, lack of energy, lack of motivation, low morale, fear avoidance and co-morbid conditions. Most took light exercise, such as walking, gardening, tai chi and pilates.

### Interviews

Data from interviews paralleled the survey results, indicating the need for education and interventions to address lifestyle changes, polypharmacy and strategies to manage multiple conditions. Individuals were also asked how they would like services to develop in future to help their co-morbidity management better.

### Access to health care

Most preferred to access care from multiple specialists. Some felt strongly that their general practitioner (GP) should be the main co-ordinator, but others felt that GPs did not have time for complex or multiple problems or had felt trapped because their GP/hospital had suggested review by each other. A number of individuals expressed frustration in not receiving timely access to health care and were affected by poor communication between the professionals supervising various aspects of their health care. Some individuals suggested that more time is needed for multiple problems and that there is a need for better oversight of the individual rather than separate conditions, because a single approach does not fit all.

### Awareness and impact of co-morbidities, themes and quotes

**Fig. 1:**
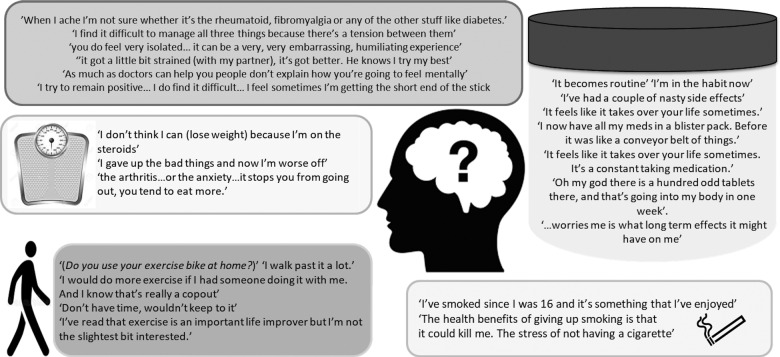
Quotes relating to awareness and impact of co-morbidities, polypharmacy and lifestyle

Quotes can be found in Fig. 1. Individuals described how multiple conditions were burdensome, caused confusion, and they were often unsure which condition was flaring. They were given opposite advice from different specialists. Many indicated that they were unaware that multiple conditions and drugs were interrelated or interlinked (e.g. CSs leading to diabetes). Some individuals described how they had to find their own management strategies by setting small goals and targets to manage Charlson co-morbidities and polypharmacy. Individuals described how co-morbidity had a significant impact on their family life, psychosocial life, employment and quality of life, in particular in the older age group.

### Polypharmacy and quotes

Quotes can be found in Fig. 1. Our results revealed that understanding of polypharmacy is limited in most individuals. Some did not know why they were prescribed so many drugs and were unsure whether the multiple pills were effective. Many expressed concerns about polypharmacy over time and the possible negative impacts on their health. Given that different medications ran out at different times, individuals often had difficulty in remembering to order medications or renew their prescription. Some of them used strategies such as an alarm for warfarin and MTX, whereas others relied on pill boxes, family members and diaries.

### Lifestyle and quotes

Quotes can be found in Fig. 1. Many individuals said they were aware of health benefits of exercise, weight loss or stopping smoking but were not sure that lifestyle change would help them personally. Fear avoidance was a commonly mentioned reason stopping individuals from being more active. They expressed lack of information on how much exercise or what type of exercise was safe for them. A few individuals reported difficulty adhering to a healthy diet, in particular individuals with diabetes or Crohn’s disease, and it took longer to do food shopping. They indicated that they did not receive any helpful advice regarding exercise or weight loss and would like guidance from their medical professionals.

## Discussion

To our knowledge, this is the first study to have explored perspectives on co-morbidities from the viewpoint of the individual suffering with them. We found substantial co-morbidities in people with inflammatory arthritis, most commonly hypertension, pulmonary disease, diabetes mellitus, ischaemic heart disease and cancer. These conditions affected their health as much as their arthritis. Issues raised were the negative impacts on quality of life, deterioration of physical function, strain on relationships and social life, helplessness, multiple appointments with different specialties, lack of clarity, psychological effects and employment issues.

Multimorbidity is reported in many chronic conditions, including chronic renal disease, with a similar lack of awareness that the index condition is associated with additional conditions [17, 18]. A review of the experience of individuals of coping with multimorbidity found similar themes to our survey, namely the need for multiple contacts with different health-care providers, negative emotions, polypharmacy and coping in the social context [19]. Our study echoes the findings of previous studies, but in addition we highlight a wider problem that health professionals face in providing effective information on co-morbidity and providing holistic management, particularly in relatively short appointments at 6- to 12-month intervals. There are international recommendations about specific co-morbidities (e.g. cardiovascular risk in RA [20]), but there are no recommendations regarding the overall management of co-morbidities except for the overarching principle that such cases need a tailored approach. It is difficult to have a single model for assessment and management of co-morbidities, and we therefore need services to be delivered by a multidisciplinary team with representation of wide-ranging skills, with clear and easy lines of communication between all parties involved, as indicated by individuals in this study. Multimorbidity is likely to increase over time and could pose considerable challenges to health systems and economies not equipped to care for complex conditions. We need to develop new models of care and better approaches to clinical management of individuals with co-morbidities.

Participants in this study were divided in their views with regard to who should provide holistic management. In the UK, annual reviews and monitoring for co-morbidities are recommended by the National Institute for Health and Care Excellence [21], thus combining disease control and co-morbidity management as part of the daily practice in inflammatory arthritis. Despite 66% of participants self-reporting that they had moderate to severe disease, 68% also reported that their treatments were effective. This represents the major improvements in modern rheumatology clinics following the treat-to-target initiatives. Several studies have found that holistic care, patient-centred information and communication skills of nurses have improved outcomes [22–25]. Annual screening for specific co-morbidities, such as cardiovascular risk, diabetes mellitus and osteoporosis, using validated tools can also be delivered within primary care. One of the concerns identified by individuals in our study was the lack of available information on the management of co-morbidities. Current guidelines from EULAR [20] state that the best care includes explicit patient involvement in treatment decisions, but some individuals in this study felt that they were insufficiently involved in the decision-making process.

We have identified that most participants reported low levels of physical activity, the reasons for which were multifactorial. Reduced physical activity in RA is associated with disease activity, but also with obesity, poor mental health and patient perception of disease [26–28]. We recommend the presence of a physiotherapist and an occupational therapist in a multidisciplinary team, which might allow these issues to be addressed in an efficient and expedient manner. Traditionally, these roles have been under-resourced, and we feel that it is crucial for every rheumatology department to be staffed adequately with these crucial resources.

Participants in our cohort were not aware of the dangers of polypharmacy and drug or disease interactions. Polypharmacy was associated with higher HAQ scores and increased risk of adverse events [29]. Medication reviews, vigilance for drug interaction and de-escalation of medication could be handled by a hospital or community pharmacist. There are tools such as START (Screening Tool to Alert doctors to Right Treatment) to evaluate a patient’s medication [30] and STRIP (Systematic Tool to Reduce Inappropriate Prescribing) to manage prescribing and de-prescribing, which also incorporates patient preferences [31]. These tools could be incorporated into clinical decision support systems to improve medication-related problems.

Our study also has limitations that we recognize. We used a non-validated questionnaire, which did not allow calibration of responses to any available metric. The exploratory single-group design and lack of control group might have introduced bias.

## Conclusions

This study has highlighted the interplay of co-morbidities with inflammatory arthritis, which leads to a deterioration in the quality of life, substantial disability and polypharmacy concerns. Individuals express the need for better education, co-ordination and communication across specialties, longer appointments and timely access to health-care resources. Services focused on the needs of these individuals are imperative for improving quality of life.

## Supplementary Material

rkaa076_Supplementary_Data
