# Supplementary material for: Patient perceptions of co-morbidities in inflammatory arthritis
Source: Rheumatol Adv Pract. 2021 Jan 11;5(1):rkaa076. doi: 10.1093/rap/rkaa076 (PMC7884022; doi:10.1093/rap/rkaa076)
Supplement: rkaa076_Supplementary_Data [file rkaa076_supplementary_data.zip › RAP 20-102 Supplementary Data S2.docx]

*Supplementary Data S2. Interview questions*

**Interview questions**

1. What effects do the multiple conditions have on your quality of life?
2. How do you manage? What strategies do you use to manage multiple conditions?
3. What do you understand by multiple medications? Explore Compliance issues ,difficulties and patient experience of taking multiple pills
4. Do you find it difficult to motivate yourself to take up lifestyle changes and what would help? Explore barriers
5. What services or help would you like to see in future to manage your multiple conditions?
6. Tell us how you think multiple conditions could be managed? Who should oversee treatment of your multiple conditions? GP/Rheumatologist/Other specialist?
