# Supplementary material for: Patient perceptions of co-morbidities in inflammatory arthritis
Source: Rheumatol Adv Pract. 2021 Jan 11;5(1):rkaa076. doi: 10.1093/rap/rkaa076 (PMC7884022; doi:10.1093/rap/rkaa076)
Supplement: rkaa076_Supplementary_Data [file rkaa076_supplementary_data.zip › RAP 20-102 Supplementary Data S3.docx]

*Supplementary Data S3. Themes and quotes*

**Theme 1: Ilustrative Quotes relating to awareness and impact of CC**

| Awareness | Impact |
| --- | --- |
| ’When I ache I’m not sure whether it’s the rheumatoid, fibromyalgia or any of the other stuff like diabetes. It does get a bit overwhelming’ | It affects me with the grandchildren. I can’t get into rough and tumbles like I’d like to’ |
| ‘I do feel a lot of the time you’re not believed… why on earth would I make it up. Not knowing how one thing is affecting something else and just having people believe you’ | I’ve got a very understanding husband who helps a lot but it’s aggravating’ |
| ‘I find it difficult to manage all three things because there’s a tension between them’ | ‘If it’s a bad day in my head it’s going to be a bad day in my body’ |
| ‘I’m very strong willed and I won’t let it(*rheumatoid)* stop me doing things. But the Crohn’s did’ | ‘you do feel very isolated sometimes and it can be a very, very embarrassing, humiliating experience’ |
| ‘As much as doctors can help you with your medications…people don’t explain how you’re going to feel mentally… my head was all over the place’ | Yeah, they’re limiting. They make it so that I have to either stop doing it or do it in a different way or not do it at all. |
| ‘tried just to continue with life as best I can’ | ‘my confidence. I used to socialise a lot, don’t do any of that anymore. I can’t work; I can’t stand for that long. I struggle with just about everything’ |
|  | Generally, it’s just having the tools or using objects to do it in a different way or using my husband to do it in a different way. So, there’s ways around most things. |
|  | ‘I think I probably argue with my husband more than I should’ |
|  | ‘’it got a little bit strained(*with my partner*), it’s got better. He knows I try my best’ |
|  | ‘My wife and daughter do all the carrying, it’s not really fair on them, I’d like to do more |
|  | ‘I can’t just get up and go and do what I want to do. There’s not always people around to do things with me and like I have this real issue about doing things on my own. So like the kids have moved back in, so like they help like with the housework and different things |
|  | ‘(*I feel*) Awful, because it’s my, not my job but I’m the mum and I’m the person that does the cleaning of the house, the washing, the putting out, so when I have to delegate things, it’s like I shouldn’t have to because it’s my role… when I can’t do it, it gets very, very frustrating. Very frustrating.’ |
|  | ‘It’s all making, having a slowing effect, slowing everything down’ |
|  | ‘I try to remain positive… I do find it difficult… I feel sometimes I’m getting the short end of the stick’ |

**Theme 2 : Quotes relating to Polypharmacy**

| Polypharmacy | Side effects /Long term effects |
| --- | --- |
| ‘They’re in the box… I order them… I know the first 5 in the morning and the last six in the evening.’ | ‘I’ve had a couple of nasty side effects’ |
| ‘It becomes routine’ ‘I’m in the habit now’ | I’m still tired, I still feel sick. I am just not 100% sure exactly what they are all doing’ |
| ‘It feels like it takes over your life sometimes. It’s a constant taking medication’ | ‘…worries me is what long term effects it might have on me’ |
| ‘I now have all my meds in a blister pack. Before it was like a conveyor belt of things. My daughter in law prompts me to make sure I’ve had them.’ | ‘’are any of these tablets actually doing what I want them to do. Because I’m still in pain, |
| ‘It’s just remembering. I’ve got an alarm set on me mobile to remind me’ |  |
| It is a bit boring sometimes. |  |
| I find reordering them is a bit of a bind because they all seem to expire at different times |  |
| ‘Oh my god there is a hundred odd tablets there, and that’s going into my body in one week’. |  |
| I think it would help if it was explained because when you, when I read the literature that comes with the medicine, sometimes it’s, I suppose, it’s almost frightening really |  |

**Theme 3 : Quotes relating to Lifestyle**

| Life style | Barriers |
| --- | --- |
| I believe the health benefits(*of exercise)*, but I don’t think it’s going to improve my health’ | ‘I cannot lead an active life due to COPD’ |
| ‘I’m not the slightest bit interested in doing any exercise’. My energy levels are low and my wife’s always saying energy makes energy, but I can’t be bothered’ | ‘I’ve been told to do exercise… but I get so out of breath’ |
| ‘(*Do you use your exercise bike at home?*)’ ‘I walk past it a lot. Yeah. It’s painful’ | ‘Don’t have time, wouldn’t keep to it’ |
| ‘You’ve still got to push yourself to do something’ ‘It’s to make me more mobile’ | ‘I don’t think I can(lose weight) because I’m on the steroids’ |
| ‘I would do more exercise if I had someone doing it with me. And I know that’s really a copout itsn’t it?’‘… | ‘Because of the arthritis…or the anxiety…it stops you from going out, you tend to eat more.’ |
| How far to push myself. It’s hard because I used to go to the gym two, three times a week. | I only used to smoke two a day because I couldn’t get out of bed. Now I will smoke 12, 15 a day because I’m more mobile. |
| ‘I know I need to exercise more. I know that but I just can’t do it when I hurt’ |  |
| ‘I’ve read that exercise is an important life improver but I’m not the slightest bit interested.’ |  |
| ‘I can do food shopping, and he would rather go to the chip shop’ |  |
| ‘I find it very difficult to change things, to cope… I wasn’t like this before I became ill… so I gave up the bad things and now I’m worse off.’ |  |
| ‘I try to eat a balanced diet because I know that’s important’ |  |
| ‘the sugar levels I keep low and I try to keep the fat levels low. You’re constantly looking at the labels. Shopping takes a bit longer’ |  |
| ‘it boils down to you, or me. If I was told, I must lose some weight then I would lose weight’ |  |
| ‘I’ve smoked since I was 16 and it’s something that I’ve enjoyed’ |  |
| ‘The health benefits of giving up smoking is that it could kill me. The stress of not having a cigarette’ |  |
