# Supplementary material for: Patient perceptions of co-morbidities in inflammatory arthritis
Source: Rheumatol Adv Pract. 2021 Jan 11;5(1):rkaa076. doi: 10.1093/rap/rkaa076 (PMC7884022; doi:10.1093/rap/rkaa076)
Supplement: rkaa076_Supplementary_Data [file rkaa076_supplementary_data.zip › RAP 20-102 Supplementary Tables.docx]

**Supplementary Table S1: Demographics and cohort characteristics**

| Age, mean  Median  < 40 yrs  40- 60 yrs  60 – 80 yrs  >80 yrs | 65.5 years  69 years  4, 3%  44, 30%  84, 58%  14,10% |
| --- | --- |
| Sex  Female  Male | 82, 56%  64, 44% |
| Marriage status  Married  Widowed  Divorced  Single  Missing  Cohabiting  Separated | 81, 56%  22, 15%  12, 8%  11,8 %  17, 12 %  2, 1.4%%  1, 0.7% |
| Smoking  Current  Ex- Smoker  Never  Missing | 20,14 %  75,52 %  50, 34%  1, 0.7% |
| Education  GCSE  A level  Degree  Post Graduate  Missing  None  Diploma | 60, 41%  14, 10%  18, 12%  6 ,4 %  17,12 %  25, 17%  6, 4% |
| Employment status  Full time  Part time  Retired  Unemployed  Missing | 28, 19%  12, 8%  85, 58%  19, 13%  2, 1.4% |
| Have you had to cut down hours or change jobs?  N/A  No  Yes  Missing | 49, 34%  63, 43%  31, 21%  3, 2% |

Data is presented as number and percent. Age presented as mean & median.

**Supplementary Table S2: Cohort characteristics**

| Charlson comorbidity  1  >/=2  >4 | N= 146  50, 35%  69, 48%  25, 17% |
| --- | --- |
| Common Comorbidities  Pulmonary disease  Hypertension  DM  Cancer  IHD  Depression  TIA | N = 146  49(34%)  75(51%)  35(24%)  20(14%)  21(14%)  19(13%)  11(8%) |
| How long have you been suffering from rheumatic disease?  < 1 yr  1- 3 yrs  3 - 5 yrs  5 - 10 yrs  > 10 yrs | 2, 1.4%  20, 14%  21, 14%  31, 21%  72 ,49 % |
| How would you rate the degree of severity of your rheumatic condition?  Very low  Low  Low - moderate  Moderate  Moderate – severe  Severe  Very severe | 7, 5%  30 ,21 %  1, 0.7%  61 ,42%  3, 2%  35, 24%  9, 6% |

Data is presented as number and percent. DM=diabetes, TIA= transient ischaemic attack, IHD=Ischaemic heart disease, N/A= not applicable

**Supplementary Table S3: Awareness and impact of comorbidities**

| **Do multiple conditions affect your health as much your arthritis?** | |
| --- | --- |
| Yes  No  Missing | 77, 53%  67, 46%  2, 1.4% |
| **What information or advice did you receive about your multiple conditions?** | |
| No information  Minimal  Detailed  Too much  Not sure  Missing | 27,19%  34,23 %  70,48%  0  14,10%  1,0.7% |
| **Are you aware that some of your conditions could be related to your meds or a complication of your arthritis?** | |
| Yes  No  Missing  Not sure | 70, 48%  72, 50%  2, 1.4%  2, 1.4% |
| **Do you need help from family members/carers?** | |
| Yes?  No | 82,56%  64,44% |
| **To what extent do you depend on them?** | |
| Daily  Twice /week  Once /week  Infrequent  Independent  Missing | 50, 34%  13, 9%  9, 6%  16, 11%  50, 34%  8, 5.5% |

Data is presented as number and percent.

**Supplementary** **Table S4: Current therapy, efficacy and patient involvement in treatment decisions**

| **Which medications are you using to control your arthritis?** | |
| --- | --- |
| NSAID – 48(33%), 73(50%) other analgesics  Monotherapy – 80/146(55%), Combination – 17(11.7%), triple therapy – 7(5%), biologics – 41(28%), GC- 32(22%), Non arthritic meds 103(71%), missing data – 19(13%) on non-arthritic meds | |
| **To what extent were you involved in the choice of your therapy /drug?** | |
| Not at all  Just a little  Somewhat  Very much  Missing | 38, 26%  35, 24%  32, 22%  39, 27%  2, 1.4% |
| **How would you rate your current treatment?** | |
| Improved Remarkably  Improved Somewhat  Didn’t make any difference  Improved arthritis but trouble side effects  Improved but need to take meds to Counteract side effects  Worsened my arthritis  Missing | 43, 30%  57, 39%  15, 10 %  17, 12%  7, 5%  6, 4 %  2, 1.4% |
| **Do your doctors advise on arthritis meds at regular follow up appointments and discuss the benefits and risk of each treatment?** | |
| Yes  No  Missing | 116, 80%  28, 19%  2, 1.4% |
| **In general, what percentage of the time do you take your medication?** | |
| 1. Arthritis   0%  10%  50%  70% -99%  100%  Missing  b. Other medications  5%  20%  50%  60 - 99%  100 %  Missing | 4, 3%  3, 2%  5, 4%  7, 5%  122, 84%  5, 4%  2,1.4%  1, 0.7%  7, 5%  9, 6%  121, 83%  6, 4% |

Data is presented as number and data. NSAID = nonsteroidal anti-inflammatory drugs, GC= glucocorticoids

**Supplementary Table S5: Lifestyle advice and benefits of life style changes**

| **Were you offered patient education seminars/groups? *** | |
| --- | --- |
| Yes  No  Not sure  Missing | 37, 25%  106, 73%  1, 0.7%  2, 1.4% |
| **Smoking is associated with poor control of arthritis, reduced response to treatment and development of comorbidities? Were you offered smoking cessation?** | |
| Yes  No  Don’t smoke  Missing | 17, 12%  24, 17%  103, 71%  2, 1.4% |
| **Have you been to smoking cessation clinics?** | |
| Yes  No  No motivation  Gave up on own  Don't smoke  Missing | 7, 5%  33, 23%  1, 0.7%  2, 1.4%  95, 65%  8, 6% |
| **High BMI can cause premature damage to the joints, increases risk of DM/hypertension and heart disease. Did you receive any advice about your weight?** | |
| Yes  No  Not overweight  Missing | 46, 32%  43, 30%  55, 38%  2,1.4% |
| **Alcohol can interfere with meds; can affect liver and cause heart disease, liver problems and stroke. What advice did you receive about alcohol intake?** | |
| No advice  Some advice  Extensive advice  Don’t drink  Missing | 17, 12%  49, 34%  9, 6%  60, 41%  11, 8% |
| **Exercise has many benefits on health. Did you receive any advice?** | |
| Yes  No  Minimal  Missing | 95, 65%  49, 34%  1, 0.7%  1, 0.7% |
| **How many minutes do you exercise in a week?** | |
| 30mins  30 - 120 mins  > 120 mins  > 240  None  missing | 5, 4%  28, 19%  35, 24%  41, 28%  31, 21%  6, 4% |
| **If no exercise, please state reasons?** | |
| Pain, fatigue, Lack of energy, lack of motivation, busy at work, low morale, Pain, Body habitus and other medical conditions. | |

Data is presented as number and percent. BMI = body mass index, DM =diabetes mellitus.

*they may have been offered face to face education but it was difficult to know how much information was retained.
