# Supplementary material for: Patient perceptions of co-morbidities in inflammatory arthritis
Source: Rheumatol Adv Pract. 2021 Jan 11;5(1):rkaa076. doi: 10.1093/rap/rkaa076 (PMC7884022; doi:10.1093/rap/rkaa076)
Supplement: rkaa076_Supplementary_Data [file rkaa076_supplementary_data.zip › RAP 20-102 Supplementary Data S1.doc]

*Supplementary Data S1. Questionnaire*

Questionnaire

| Recruiting site |  |
| --- | --- |

Principal Researcher: GMK

Version 3.1. 28/03/2018

Prospective observational non-interventional study to assess patient perception of comorbidities in arthritis patients

Questionnaire

**Confidential**

1. Do you have any of those conditions listed in the box ………………
2. Age	……………………………….
3. Sex	Male ……	Female ……….
4. Marriage Status: Married/Divorced/ Widowed/ Separated/ Cohabiting/ Single
5. Height…………. Weight …………..
6. Smoking history

Current	1 - 5 years ……. 5 -10 yrs……. > 10 yrs ……..

Smoker gave up < 12 months ……….. 1- 5 yrs……… > 5 yrs …..

Never……

7. Alcohol: Do you drink alcohol? If so, how many units a week?

(To provide a chart)

8. Your highest level of education?

GCSE/A level or equivalent	...………………………

Degree or equivalent	…………………………..

Postgraduate qualification	…………………………..

9. Employment status	Full time/part time/retired//manual/semi-manual/sedentary office Job/Not employed
10. Have you had to cut down the hours or change Jobs?

Yes ……………….. No ……………………

11. How long have you been suffering from your rheumatic disease?

Less than one year	……………………

1–3 years	……………………

3–5 years	……………………

5–10 years	……………………

More than 10 years	……………………

12. How would you rate the degree of severity of your rheumatic condition?

Very low	…………………

Low	…………………

Moderate	…………………

Severe	……………………

Very severe	………………….

**Awareness and impact of comorbidities**

13. Do multiple conditions affect your health as much as your arthritis?

Yes ………………………	No ……………………….

14. What information or advice did you receive about your multiple conditions?

No information	……………………….

Minimal Information	……………………….

Detailed Information	……………………….

Too much information	……………………….

Not sure	………………………

15. Are you aware that some of your multiple conditions could be related to your medications or are a complication of your arthritis?

Yes	……………….	No	………………………

If so, which conditions?………………………………

16. Do you need help from family members/carers?(e.g. Shopping, Physical, emotional, financial)

Yes ……………….. No……………

17. To what extent do you depend on them?

Daily/more than once a day/ Twice a week/ once a week/ infrequent/ Independent

**Current treatments**

18. Which medicines are you using to control your disease? Tick all that apply

1. Paracetamol

2. Anti-inflammatory drugs (NSAID) e.g. Ibuprofen/Naproxen/diclofenac

3. Methotrexate/Sulphasalazine /Hydroxycholorquine/Leflunomide

4. Prednisolone tablets	current dose ……

5. Intramuscular Steroid injections, how many in the last 2 years? ……………..

6. Joint injections, how many in the last 2 years?........................

7. Biologic therapies please specify which one ……………….

Other medications for e.g. Blood pressure medications, please state

………………………………………………………………..

……………………………………………………………….

……………………………………………………………….

19. To what extent were you involved in the choice of your therapy/drug?

Not at all	………………………..

Just a little	………………………..

Somewhat	……………………….

Very much	……………………….

20. Successful treatment of arthritis should reduce joint pain and swelling, slow down joint damage, avoid disability and improve quality of life. How would you rate your current treatment? Tick all those apply

Has improved my condition remarkably

Has improved it somewhat

Did not make any difference

Has improved my arthritis but I have troublesome side effects

Has improved my arthritis but I need to take other medications to counteract side effects

Has worsened my arthritis

21. Do your doctors advise on arthritis medications at regular follow up appointments and discuss the benefits and risks of each treatment?

Yes ………………….	No ……………………..

22. In general what percentage of the time do you take your medication? a)arthritis medication 0……………………………100%

b) other medications	0…………………………..100%

**Lifestyle**

23. Were you offered Patient education seminars/groups,

Yes ………………..	No …………………

24. Smoking is associated with poor control of arthritis, reduced response to treatment and development of comorbidities? Were you offered smoking cessation

Yes/No/I do not smoke

25. Have you been to smoking cessation clinics?

Yes/No/I do not smoke

26. High BMI or weight can cause premature damage to the joints, increases the risk of diabetes, hypertension and heart disease. Did you receive any advice about your weight?

Yes/No/I am not overweight

27. Alcohol can interfere with medications, can affect the liver and can cause heart disease, liver problems and stroke. What advice did you receive about alcohol intake?

No advice/Some advice/Extensive advice/I do not drink alcohol

28. Exercise has many benefits on health. Did you receive any advice?

Yes ………………. No ………………………

29. How many minutes do you exercise in a week? ………………

and What exercise do you take?

……………………………………………………………………………..

30. If No exercise please state reasons?

…………………………………………….

Thank you for taking the time to complete this questionnaire. Please return it to the health professional or post it back in the freepost envelope enclosed.

✂………………………………………………………………………………………………………………………………

We are also conducting an interview study to help us understand more about people’s views. The interview will be between 30-45 minutes. This will take place either at the hospital or alternatively, it could be done over the telephone. If you are willing to take part in an interview, please leave your contact details and a researcher may contact you (please note not everyone will be contacted):

Name:

Tel:..........................	Email: ……………………
